# OVATE Family Protein 8 Positively Mediates Brassinosteroid Signaling through Interacting with the GSK3-like Kinase in Rice

**DOI:** 10.1371/journal.pgen.1006118

**Published:** 2016-06-22

**Authors:** Chao Yang, Wenjin Shen, Yong He, Zhihong Tian, Jianxiong Li

**Affiliations:** 1 University of Chinese Academy of Sciences, Key Laboratory of South China Agricultural Plant Molecular Analysis and Genetic Improvement, and Guangdong Provincial Key Laboratory of Applied Botany, South China Botanical Garden, Chinese Academy of Sciences, Guangzhou, China; 2 College of Life Science, Yangtze University, Jingzhou, China; 3 Hubei Collaborative Innovation Center for Grain Industry, Yangtze University, Jingzhou, China; University of California Berkeley, UNITED STATES

## Abstract

*OVATE* gene was first identified as a key regulator of fruit shape in tomato. OVATE family proteins (OFPs) are characterized as plant-specific transcription factors and conserved in *Arabidopsis*, tomato, and rice. Roles of OFPs involved in plant development and growth are largely unknown. Brassinosteroids (BRs) are a class of steroid hormones involved in diverse biological functions. OsGKS2 plays a critical role in BR signaling by phosphorylating downstream components such as OsBZR1 and DLT. Here we report in rice that *OsOFP8* plays a positive role in BR signaling pathway. BL treatment induced the expression of *OsOFP8* and led to enhanced accumulation of OsOFP8 protein. The gain-of-function mutant *Osofp8* and *OsOFP8* overexpression lines showed enhanced lamina joint inclination, whereas *OsOFP8* RNAi transgenic lines showed more upright leaf phenotype, which suggest that *OsOFP8* is involved in BR responses. Further analyses indicated that OsGSK2 interacts with and phosphorylates OsOFP8. BRZ treatment resulted in the cytoplasmic distribution of OsOFP8, and bikinin treatment reduced the cytoplasmic accumulation of OsOFP8. Phosphorylation of OsOFP8 by OsGSK2 is needed for its nuclear export. The phospphorylated OsOFP8 shuttles to the cytoplasm and is targeted for proteasomal degradation. These results indicate that OsOFP8 is a substrate of OsGSK2 and the function of *OsOFP8* in plant growth and development is at least partly through the BR signaling pathway.

## Author Summary

OVATE family proteins (OFPs) are characterized as plant-specific transcription factors and mainly function in affecting fruit shape, but the molecular mechanisms by which they function are largely unknown. Rice genome contains 31 OFPs, the roles of these OsOFPs involved in plant development and growth are not understood. Brassinosteroids (BRs) are a class of steroid hormones involved in diverse biological functions. Here we report in rice that *OsOFP8* plays a positive role in BR signaling pathway by interacting with OsGKS2, a negative regulator in BR signaling pathway. Our results shed light on studying the functions of OFPs and provide a chance to explore the new components of BR signaling pathway.

## Introduction

*OVATE* gene was first cloned in tomato and demonstrated to encode a hydrophilic protein with putative bipartite nuclear localization signal, and a C-terminal domain of approximate 70 amino acids which is designated as the OVATE domain and conserved in tomato, *Arabidopsis*, and rice [1–3]. As a plant-specific transcription factor family, OVATE family proteins (OFPs) control multiple aspects of plant growth and development [2, 4–6]. Sequence analysis showed that there are 18 *OVATE* genes in the *Arabidopsis* genome [2, 5–7]. AtOFP1 was shown to function as an active transcriptional repressor to suppress cell elongation [2]. *Arabidopsis* plants overexpressing *AtOFP1* exhibited abnormal morphological phenotypes because AtOFP1 suppresses the expression of *AtGA20ox1*, the key gibberellin (GA) biosynthesis enzyme gene [2]. AtOFP4 was reported to interact with KNAT7 (Knotted1-Like Homeodomain Protein 7) *in planta*, this interaction enhances KNAT7’s transcriptional repression activity and regulates the secondary cell wall formation [5]. AtOFP5 is required for normal embryo sac development in *Arabidopsis* by suppressing the activity of BELL-KNOX TALE complexes [7].

In rice, there are 31 putative OFPs identified in the genome [3]. Although increasing evidence in *Arabidopsis* demonstrates that AtOFPs participate in multiple aspects of plant growth and development by regulating the transcriptional levels of target genes, little is known about the function and action mode of OsOFPs in rice.

Brassinosteroids (BRs) are a class of plant-specific steroidal hormones that are structurally related to animal and inset steroids. As a group of growth-promoting steroid hormones, BRs play pivotal roles in promoting cell expansion and division, regulating senescence, male fertility, fruit ripening, and modulating plant responses to various environmental signals [8]. Extensive studies in *Arabidopsis* have identified a nearly complete BR signaling pathway starting with BRI1 (Brassinosteroid insensitive 1) as the cell membrane receptor which perceives and binds to BR [9], then initiating a phosphorylation-mediated cascade involving BSK1 (BR-signaling kinase 1), BSU1 (BRI1 suppressor 1), BIN2 (BR-insensitive 2), and PP2A (Protein phosphatase 2A), and finally transducing the extracellular signal to the transcription factor BZR1 (Brassinazole resistant 1) [10–13]. In this signaling pathway, BIN2 acts as a negative regulator that interacts with and phosphorylates BZR1 to inhibit its function, thereby blocking BR signaling [14, 15]. BIN2 can also phosphorylate Auxin Response Factor 2 (ARF2), resulting in the inhibition of the DNA binding activity of ARF2, thus promoting downstream auxin responses [16]. In addition, BR regulates stomatal development through BIN2-mediated phosphorylation of YDA, a mitogen-activated protein kinase kinasekinase (MAPKKK) [17, 18]. These studies indicated that BIN2 acts as a multi-tasker in diverse cellular signal transduction pathways [19].

In rice, OsGSK2 is the counterpart of *Arabidopsis* BIN2, and acts as a negative regulator to mediate BR signaling [20]. The phosphorylated form of OsBZR1 was increased in *OsGSK2* overexpression plants, and decreased in *OsGSK2* RNAi plants, suggesting that OsGSK2 mediates BR signaling through OsBZR1 [20]. In addition to OsBZR1, two other proteins in rice have been found as substrates for OsGSK2. DLT (Dwarf and Low-Tillering), encoding a GRAS-family protein, is a direct target of OsGSK2 and functions similarly to OsBZR1 [20–22]. In contrast to DLT, OsLIC (LEAF and TILLER ANGLE INCREASED CONTROLLER), another substrate of OsGSK2, acts as an antagonistic transcription factor of OsBZR1 and plays a negative role in BR signaling [23]. These studies suggested the vital role of OsGKS2 in BR signaling.

We report here the characteristics of *OsOFP8*, a member of OVATE family protein genes in rice. The gain-of-function mutant *Osofp8* and *OsOFP8* overexpression transgenic lines showed enhanced lamina joint bending, whereas *OsOFP8* RNAi lines showed upright leaves and tight architecture. Further analysis revealed that OsGSK2 interacts with and phosphorylates OsOFP8, and phosphorylated OsOFP8 shuttles to the cytoplasm and is targeted for the proteasomal degradation.

## Results

### Increase in *OsOFP8* expression results in lamina joint bending phenotype

**Fig 1:**
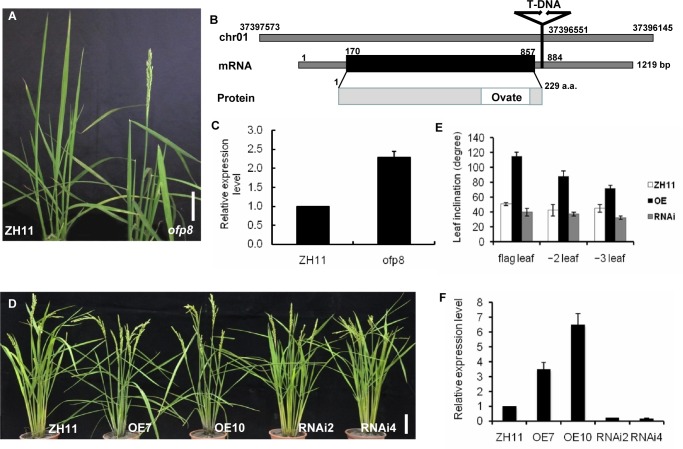
Phenotypes of *Osofp8* mutant, *OsOFP8*-OE plants, and *OsOFP8*-RNAi plants. (A) Gross morphology of *Osofp8* mutant compared with the wild-type (ZH11). Bars = 10 cm. (B) Schematic structure of *OsOFP8* gene. *OsOFP8* is an intronless gene encoding a protein of 229 amino acids. The T-DNA insertion site is located at the 27 bp downstream of the stop codon of the *OsOFP8* gene and indicated by an insertion tag. (C) Relative transcript level of *OsOFP8* in ZH11 and *Osofp8* mutant plants. Two-week-old seedlings of ZH11 and *Osofp8* mutant plants were used for qRT-PCR analysis. *OsActin 1* was used as a control. (D) Gross morphology of *OsOFP8*-overexpression lines (OE7 and 17), *OsOFP8*-RNAi lines (RNAi2 and 4), and ZH11 plants. Bars = 10 cm. (E) Leaf inclination of the last three leaves at the heading stage of ZH11, *OsOFP8*-OE, and *OsOFP8*-RNAi lines. The leaf angles were measured in duplicate with five plants for each type of plants, and data are presented as mean ± SE. (F) qRT-PCR analysis of *OsOFP8* expression in different *OsOFP8*-OE and *OsOFP8*-RNAi lines compared with ZH11. Two-week-old seedlings were used for the analysis. *OsActin 1* was used as an internal control.

In the experiment of generation of T-DNA mutants, we identified a T-DNA insertion mutant showing lamina joint bending phenotype at the maturation stage, especially for the flag leaves (Fig 1A). To identify the gene in which T-DNA was inserted, we performed TAIL-PCR analysis [24], DNA sequence comparison showed that the T-DNA was inserted into the 3’ region of LOC_Os01g64430 at the site of 27 bp downstream of the stop codon (Fig 1B), and there is no other annotated genes in the 5.5 kb region downstream of LOC_Os01g64430 (http://rice.plantbiology.msu.edu). LOC_Os01g64430 encodes an OVATE family protein (hereafter designated as OsOFP8). To investigate the effect of T-DNA insertion on the expression of *OsOFP8*, we carried out quantitative real-time RT-PCR (qRT-PCR), which showed that T-DNA insertion causes an increase in the expression of *OsOFP8* (Fig 1C), thus T-DNA insertion generates a gain-of-function mutant *Osofp8*.

To further investigate the function of *OsOFP8* gene, we generated both *OsOFP8* overexpression and RNA-interference (RNAi) transgenic lines. *OsOFP8* overexpression lines OE7 and OE10 phenocopied the *Osofp8* mutant, showing increased lamina joint bending phenotype, by contrast, RNAi transgenic lines RNAi2 and RNAi4 showed upright leaves and tight architecture (Fig 1D). Furthermore, we examined the leaf inclination degrees of the three uppermost leaves. Compared to the wild-type (WT) plants, *OsOFP8* overexpression plants showed largely increased leaf inclination for all three uppermost leaves, and the flag leaf showed the largest inclination angle, by contrast, RNAi transgenic plants showed reduced leaf angles (Fig 1E). Expression analysis by qRT-PCR showed that *OsOFP8* expression was increased in overexpression lines and reduced in RNAi lines (Fig 1F). Tissue-specific expression of OsOFP8 was examined by qRT-PCR analysis, showing that *OsOFP8* was expressed in various tissues (S1A Fig). Native promoter of *OsOFP8* was fused to *GUS* gene to gain expression profile of *OsOFP8*, GUS activity was detected in different organs including roots, stem, leaf, lamina joint, inflorescence, and seeds (S1B–S1J Fig).

### OsOFP8 positively functions in BR pathway

**Fig 2:**
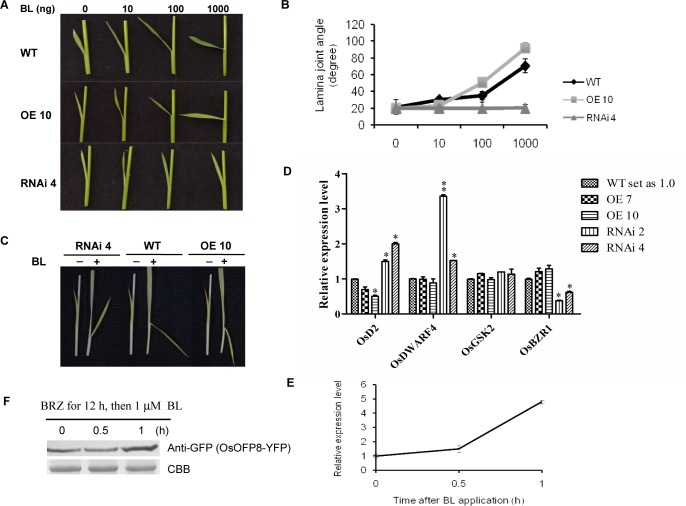
OsOFP8 is a positive regulator in BR signaling in rice. (A) Lamina joint bending response to various amounts of 24-epiBL by the micro-drop method. A drop of ethanol (1 μL) containing 0, 10, 100 or 1000 ng of 24-epiBL, respectively, was spotted onto the top of the lamina of seedlings which were germinated for 2 days and grown for 3 days at 30°C. Images were obtained after 3 days of incubation. (B) Statistical data for lamina joint bending angle assay described in (A). The leaf angle assay was performed in two biological repeats, and each repeat with five plants measured for each concentration and each type of plants. Data are presented as mean ± SE. (C) Lamina joint bending response to 1 μM 24-epiBL by the excised leaf segment method. Seeds were germinated for 2 days and then grown in the dark for 8 days at 30°C. Segments comprising of a part of the second leaf blade, lamina joint, and leaf sheath were floated on distilled water for 24 h and then incubated in 1 μM 24-epiBL for 48 h in the dark. The experiments were performed in triplicate with similar results. (D) qRT-PCR analysis of the expression level of *OsD2*, *OsDWARF4*, *OsGSK2*, and *OsBZR1* genes in WT, *OsOFP8*-OE, and *OsOFP8*-RNAi plants. Two-week-old seedlings were used for analysis, which was performed in three biological repeats, each with three seedlings. Data represent mean ± SE, *, p < 0.05; **, p < 0.01, t-test. *OsActin 1* was used as a control. (E) qRT-PCR analysis of the expression level of *OsOFP8* under the treatment of 1 μM 24-epiBL. Two-week-old seedlings were used for analysis with two biological repeats, each repeat having three seedlings for control and treatment, respectively. Data represent mean ± SE. (F) BR signaling induces OsOFP8 protein accumulation. *Arabidopsis* protoplast cells were transfected with *OsOFP8-YFP* and treated with 10 μM BRZ for 12 hours (h). After the treatment, BRZ was washed off, and then the cells were treated with 1 μM BL and harvested at the indicated time points, the protein was extracted from the harvested cells and used for western blotting, YFP antibody was used to detect OsOFP8 protein. CBB, Coomassie Brilliant Blue.

Gain-of-function *Osofp8* mutant and *OsOFP8* overexpression lines showed obvious leaf lamina joint bending phenotype, which is a classic phenotype of BR response. We hypothesized that *OsOFP8* may be involved in BR signaling pathway. To this end, we tested the sensitivity of wild-type plants, *OsOFP8* overexpression and RNAi lines to 24-epibrassinolide (BL) in lamina joint bending experiments. BL treatment caused a dose-dependent lamina joint inclination in both WT and OE10 with the latter plants were more sensitive to BL treatment, whereas RNAi4 plants were insensitive to BL treatment (Fig 2A and 2B). To further confirm this phenomenon, lamina joint assay was performed in the dark-grown seedlings by the excised leaf segment method [22]. We observed more severe inclination of leaf angle in OE10 than in WT plants, whereas RNAi4 plants did not show too much change in leaf angle when treated with BL (Fig 2C).

To investigate the effect of *OsOFP8* on the expression of BR-related gene expression, we analyzed the expression levels of genes involved in BR biosynthesis and signaling. Rice *D2/CYP90D2* (*OsD2*) gene is involved in the last step of brassinosteroid biosynthesis [25], and *OsDWARF4/CYP90B2* functions in the rate-limiting step of brassinosteroid biosynthesis [26]. Overexpression of *OsOFP8* suppressed the expression level of *OsD2* but had little effect on the expression of *OsDWARF4*, whereas knockdown of *OsOFP8* expression by RNAi led to significantly increased expression of *OsD2* and *OsDWARF4* (Fig 2D). OsOFP8 had little effect on the expression of *OsGSK2*, a negative regulator gene in BR signaling, by contrast, the expression levels of *OsBZR1*, a positive controller in BR signaling, were increased in *OsOFP8* overexpression lines and significantly decreased in *OsOFP8* RNAi lines (Fig 2D). We also measured the expression of *OsOFP8* in BR signaling. BL treatment induced the mRNA transcript of *OsOFP8* (Fig 2E). To further investigate the effect of BR signaling on *OsOFP8* expression, we first treated the *OsOFP8*-*YFP* transfected protoplast cells with BRZ for 12 hr, and then BL was applied after the removal of BRZ. The protein level of OsOFP8 was induced by BL treatment (Fig 2F).

### OsGSK2 interacts with and phosphorylates OsOFP8

**Fig 3:**
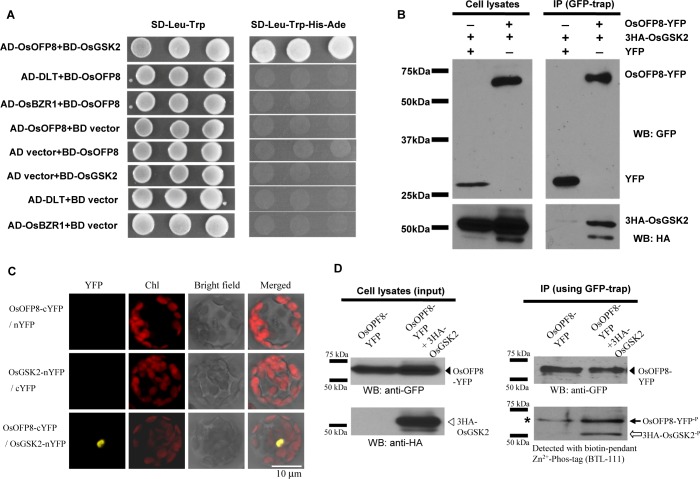
OsGSK2 interacts with and phosphorylates OsOFP8. (A) Yeast two-hybrid analysis for the interaction between OsOFP8 and OsGSK2, DLT, and OsBZR1. Co-transformed yeast clones were placed on SD dropout plates to detect the interactions. SD-Leu-Trp: synthetic complete medium lacking Leu and Trp for co-transformation detection. SD-Leu-Trp-His-Ade: synthetic complete medium lacking Trp, Leu, His and Ade for interaction detection. (B) Immunoprecipitation (IP) assay shows that OsOFP8 is in association with OsGSK2. *Arabidopsis* protoplasts expressing either YFP and 3HA-OsGSK2 or OsOFP8-YFP and 3HA-OsGSK2 were subjected to protein extraction. The Input (cell lysate) and IP were immunoblotted with indicated antibodies. WB: GFP indicates western blotting with GFP antibody. WB: HA indicates western blotting with HA antibody. (C) BiFC assay shows the interaction between OsOFP8 and OsGSK2. Chl means chlorophyll. (D) OsOFP8 phosphorylation analysis. *Arabidopsis* protoplast cells expressing *OsOFP8-YFP* only or *OsOFP8-YFP* with *3HA-OsGSK2* were subjected to protein extraction and then immunoprecipitated with either GFP antibody (WB: ant-GFP) or HA antibody (WB: anti-HA). Phosphorylation was detected with biotin-pendant Zn^2+^-Phos-tag (BTL-111). Black and white arrowheads indicate OsOFP-YFP and 3HA-OsGSK2, respectively. Black and white arrows indicate phosphorylated OsOFP8-YFP^-P^ and 3HA-OsGSK2^-P^, respectively. * represents the internally phosphorylated OsOFP8-YFP.

The different responses of *OsOFP8* overexpression and RNAi plants to BL treatment prompted us to further explore the possible functions of *OsOFP8* in BR signaling pathway. We performed yeast two-hybrid (Y2H) analysis to test the interactions between OsOFP8 and the components of BR signaling. OsGSK2, OsBZR1, and DLT are three important components in BR signaling pathway, we investigated the possible interactions between OsOFP8 and these three components. Y2H analysis showed that OsOFP8 interacted with OsGSK2, but not with OsBZR1 and DLT (Fig 3A). The interaction between OsOFP8 and OsGSK2 required the full length of OsOFP8 because neither the N-terminal nor the OVATE domain-containing C-terminal of OsOFP8 interacts with OsGSK2 (S2A and S2B Fig). The interaction between OsOFP8 and OsGSK2 was also confirmed by the coimmunoprecipitation (Co-IP) assay. The HA-tagged OsGSK2 protein (3HA-OsGSK2) and YFP-tagged OsOFP8 protein (OsOFP8-YFP) were co-transfected into *Arabidopsis* protoplast cells, the fusion protein 3HA-OsGSK2 can be immunoprecipitated by OsOFP8-YFP fusion but not by YFP protein (Fig 3B). Furthermore, BiFC assay was used to confirm the interaction between OsOFP8 and OsGSK2 (Fig 3C).

OsGSK2 protein phosphorylates proteins such as OsBZR1 and DLT with which it interacts [20]. In this scenario, we were interested to know whether OsGSK2 phosphorylates OsOFP8. To this end, we applied the biotin-pendant Zn^2+^-phos-tag and horseradish peroxidase-conjugated streptavidin method [27] to investigate the phosphorylation status of OsOFP8 when *OsOFP8-YFP* was expressed alone or co-expressed with *3HA-OsGSK2* in *Arabidopsis* protoplast cells. When *OsOFP8-YFP* was expressed alone in the protoplast cells, we only detected a faint band showing phosphorylated OsOFP8 (Fig 3D, asterisk), which is probably caused by the endogenous BIN2 of the protoplast cells. When *OsOFP8-YFP* and *3HA-OsGSK2* were co-expressed in protoplast cells, a stronger band was detected, indicating the increased phosphorylation status of OsOFP8 (Fig 3D, black arrow). The lower band showed the phosphorylated OsGSK2 (Fig 3D), which can be used as an internal reference for the system. This assay showed that OsGSK2 is able to phosphorylate OsOFP8. GSK3 kinases recognize a conserved sequence for phosphorylation (S/TXXXS/T, where S/T is serine or threonine and X is any amino acid), for example, BZR1 protein has 25 serine/threonine residues potentially phosphorylated by BIN2 [28]. Examination of OsOFP8 protein sequence revealed that there are 25 GSK3 kinase phosphorylation sites at the N-terminal region of OsOFP8 (S2C Fig), further supporting the notion that OsGSK2 phosphorylates OsOFP8.

### OsOFP8 localizes to the nucleus and the phorphorylated OsOFP8 shuttles to the cytoplasm for proteasomal degradation

**Fig 4:**
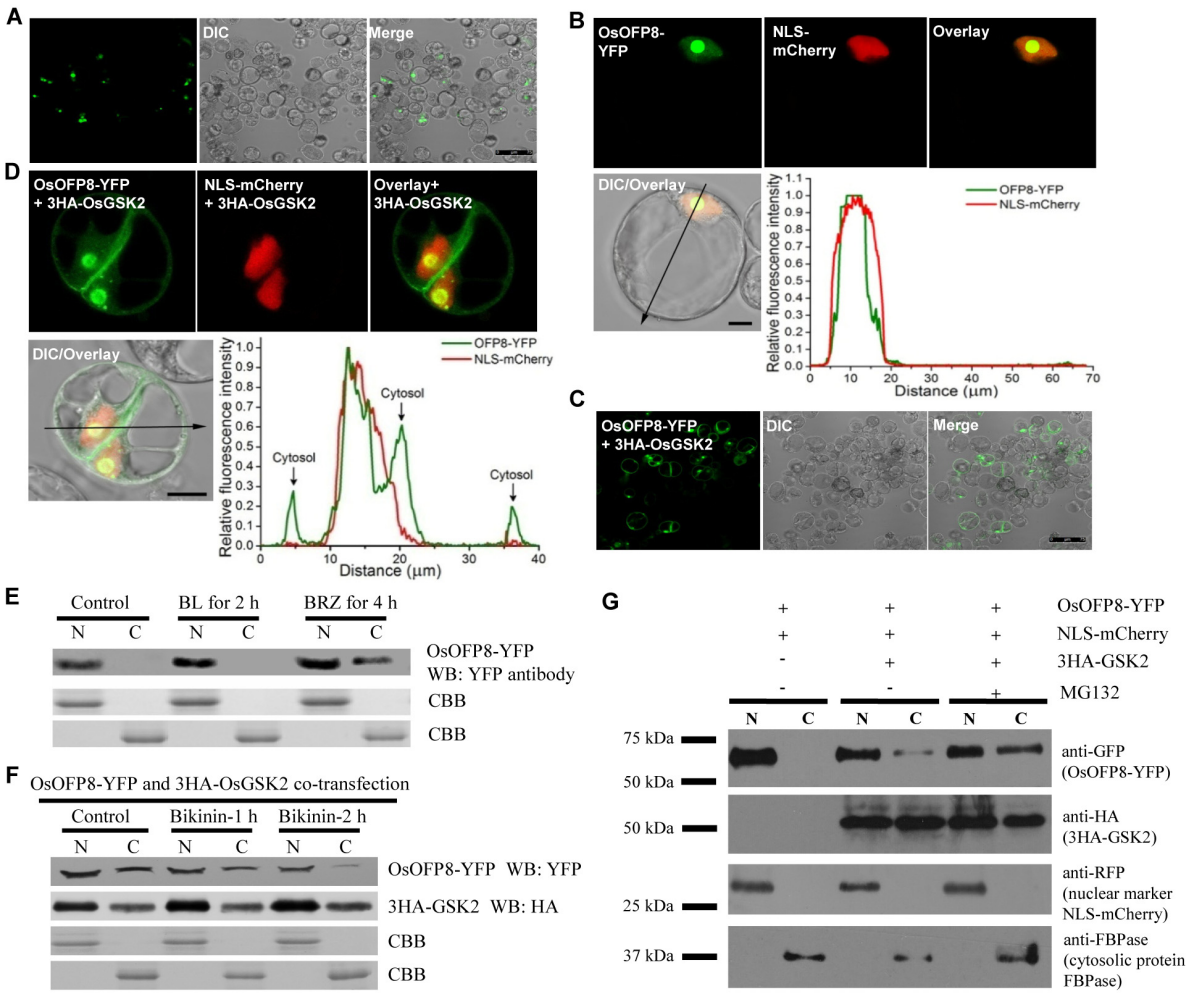
Phosphorylated OsOFP8 shuttles to the cytoplasm for proteasome-mediated protein degradation. (A) OsOFP8 locates to the nucleus. (B) A closer view of OsOFP8 distribution in the nucleus shows that OsOFP8 is predominantly located in the nucleolus. NLS-mCherry was used as a nuclear marker. The fluorescent signals were analyzed using LSM Image Browser Rel. 4.0 software, and the OsGSK2-YFP and NLS-mCherry signals match well in the nucleus (lower panel). (C) OsGSK2 leads to the nuclear export of OsOFP8. OsOFP8-YFP and 3HA-OsGSK2 were co-transfected into protoplast cells, and OsOFP8 is exported from the nucleus. (D) A closer view of the nuclear export of OsOFP8. NLS-mCherry was used as a nuclear marker. The fluorescent signal intensities of OsOFP8-YFP and NLS-mCherry were determined along the line drawn on the confocal images using LSM Image Browser Rel. 4.0 software. Different signal peaks of OsOFP8-YFP were detected in the nucleus and cytoplasm (lower panel). (E) The effect of BR and BRZ on the subcellular distribution of OsOFP8. Protoplast cells were transfected with OsOFP8-YFP, and then subjected to BL (1 μM) and BRZ (10 μM) treatment, respectively. Proteins were separately prepared from the nucleus and cytoplasm, and blotted with YFP antibody. N and C represent the nucleus and cytoplasm, respectively. WB stands for western blotting. CBB is for Coomassie Brilliant Blue. (F) Phosphorylation by OsGSK2 is needed for the nuclear export of OsOFP8. *Arabidopsis* protoplast cells were co-transfected with OsOFP8-YFP and 3HA-OsGSK2, the transfected cells were treated with bikinin (20 μM), and then harvested at the indicated time points for analysis. Proteins were separately extracted from the nucleus and cytoplasm, and then blotted with YFP and HA antibodies, respectively. N and C represent the nucleus and cytoplasm, respectively. WB stands for western blotting. CBB is for Coomassie Brilliant Blue. (G) Phosphorylated OsOFP8 shuttles to the cytoplasm for proteasomal degradation. OsOFP8-YFP combined with either 3HA-OsGSK2 or NLS-mCherry was used to transfect *Arabidopsis* protoplast cells, after incubation for 8–10 h, the cells were transferred to W5 solution with or without 10 μM MG132. The cytoplasmic and nuclear fractions from the protoplast cells were separated by centrifugation. The proteins were blotted with GFP and HA antibodies, respectively. MG132 treatment increased the amount of OsOFP8 in the cytoplasm. N and C stand for the nuclear and cytosolic fractions, respectively. NLS-mCherry and cytosolic protein FBPase were detected using anti-RFP and anti-FBPase, respectively.

To investigate the subcellular localization of *OsOFP8*, we made various OsOFP8 fusions in which YFP protein was fused to either the N-terminus or the C-terminus of OsOFP8, the fused OsOFP8 protein was transiently expressed in *Arabidopsis* protoplast cells to monitor the localization of OsOFP8. This assay showed that OsOFP8 localizes to the nucleus (Figs 4A, S3A and S3B). NLS-mCherry gene is expressed in the nucleus, the merged image of NLS-mCherry and OsOFP8-YFP showed that majority of OsOFP8 exists in the nucleolus, which was indicated by the strong fluorescence intensity in the round structure of the nucleus and seen in the differential interference contrast (DIC) images (Fig 4B). In addition, the fluorescent signals of OsOFP8-YFP and NLS-mCherry were well overlapped, further supporting the nuclear localization of OsOFP8 (Fig 4B).

To test whether the interaction with OsGSK2 alters subcellular localization of OsOFP8, we investigated the localization of OsOFP8 in the presence of OsGSK2. Co-expression with OsGSK2 clearly caused the cytoplasmic distribution of OsOFP8 (Fig 4C), and western blotting was carried out to show the presence of OsGSK2 (S3C Fig). A closer view of individual cells showed both the nuclear and cytoplasmic localization of OsOFP8 (Fig 4D, upper panel), and analysis of the fluorescent signal peaks showed that only one peak of OsOFP8-YFP was overlapped with that of NLS-mCherry, and three other peaks of OsOFP8-YFP were detected in the cytosol (Fig 4D, lower panel). These results indicate that interaction with OsGSK2 leads to the nuclear export of OsOFP8 to the cytoplasm.

To analyze the effect of BR signaling on the subcellular distribution of OsOFP8, we treated the *OsOFP8-YFP* transfected protoplast cells with BL. After BL treatment for two hours, the protein level of OsOFP8 was increased in the nucleus, and OsOFP8 was not detected in the cytoplasm (Fig 4E). When treated with BRZ, a BR biosynthetic inhibitor brassinazole, the protein level of OsOFP8 was detected both in the nucleus and in the cytoplasm, indicating that BRZ treatment altered the subcellular localization of OsOFP8 (Fig 4E). OsGSK2 interacts with and phosphorylates OsOFP8, the phosphorylation status of OsOFP8 may be required for its nuclear export. To this end, we treated the protoplast cells co-transfected by *OsOFP8-YFP* and *3HA-OsGSK2* with bikinin which inhibits the activity of BIN2 by acting as an ATP competitor. Western blotting showed that the protein level of OsOFP8 in the cytoplasm was largely reduced when the cells were treated with bikinin for two hours, indicating that phosphorylation of OsOFP8 by OsGSK2 is needed for its cytoplasmic localization (Fig 4F). Because presence of OsGSK2 induced the nuclear export of OsOFP8 (Fig 4D and 4F), we further analyzed the state of OsOFP8 and the phosphorylated OsOFP8 in the nucleus and cytoplasm, respectively. Nuclear and cytoplasmic fractions were prepared both from the protoplasts co-transfected with *OsOFP8* and NLS-mCherry and with *OsOFP8* and *OsGSK2*. In the absence of OsGSK2, OsOFP8 was only detected in the nucleus, which is consistent with the previous findings (Fig 4A and 4E). In the presence of OsGSK2, OsOFP8 was detected both in the nucleus and in the cytoplasm, but the band in the cytoplasm was much weaker than that in the nucleus (Fig 4G). When the co-transfected protoplasts were treated with MG132, a proteasome inhibitor, the intensity of the band in the cytoplasm was increased (Fig 4G), which suggests that the phosphorylated OsOFP8 is cytoplasm-localized and targeted for proteasomal degradation.

## Discussion

The *OVATE* gene was first identified as a major QTL controlling pear-shaped fruit in tomato [1, 29], and later on, studies in *Arabidopsis* show that the OVATE family proteins control multiple aspects of plant growth and development [2, 4–6]. The rice genome contains more number of OFPs than the *Arabidopsis* genome, but very few studies on OFP function in rice have been reported. We studied the function of *OsOFP8* in rice, and demonstrated that *OsOFP8* is involved in BR signaling. Elevated expression of *OsOFP8* in rice leads to increased lamina joint bending phenotype and BR hypersensitivity (Fig 2).

In *Arabidopsis*, *Atofp1-1D* is a dominant, gain-of-function mutant, which has a T-DNA inserted at the 4332 bp downstream of the stop codon of the *AtOFP1* gene and shows increased expression of *AtOFP1* [2]. *Osofp8* is also a gain-of-function mutant with T-DNA inserted in the 3’ region of *OsOFP8* gene and shows increased expression of *OsOFP8* gene (Fig 1). This coincidence of gain-of-function mutants generated by T-DNA insertion may imply a common mechanism regarding the expression regulation of OFP genes. In *Arabidopsis*, *Atofp1-1D* mutant shows reduced lengths in all aerial organs including hypocotyls, rosette leaf, inflorescence stem and floral organs. By contrast, the rice *Osofp8* mutant displays increased lamina joint inclination but does not show reduced length of aerial organs (Fig 1). Furthermore, *AtOFP1* is involved in GA signaling by repressing the expression of *GA20ox1*, a gene encoding a key enzyme in GA biosynthesis, but OsOFP8 is involved in BR signaling pathway and shows normal response to GA treatment (S4 Fig), indicating the functional diversity of these two genes in *Arabidopsis* and rice. AtOFP1 is expressed in roots, shoots, vasculatures, trichomes, and in mature flowers. Similarly, OsOFP8 is expressed in roots, shoots, and inflorescences (S1 Fig). Sequence analysis of AtOFP1 and OsOFP8 proteins also showed different functional domains. OVATE domain is the common feature for all OFP proteins, besides this, AtOFP1 contains an LXLXL motif (where L is leucine and X for any amino acid) in its OVATE region, which is not present in OsOFP8. The LXLXL motif has been shown to play an important role in repression of gene expression [2], although it only contributes marginal repression function to the AtOFP1.

*OsGSK2* is an ortholog of *Arabidopsis BIN2* gene and plays negative roles in BR signaling [20]. The expression levels of BR-biosynthesis related genes such as *OsD2* and *OsDWARF4* were increased in *OsGSK2* overexpression line and decreased in *OsGSK2* RNAi line [20]. By contrast, *OsBZR1* and *DLT* plays positive roles in BR signaling, and *OsD2* and *OsDWARF4* expression levels were induced in *OsBZR1* RNAi plants and *dlt* mutant [20, 22]. On the contrary, the expression levels of *OsD2* and *OsDWARF4* were increased in *OsOFP8* RNAi lines, and the expression of *OsD2* was reduced in *OsOFP8* overexpression lines although in these lines *OsDWARF4* did not show much change in expression when compared to its expression in wild-type plants. BL treatment (1 μM) increases *OsOFP8* mRNA transcript and the protein amount at the translational level (Fig 2E and 2F), at this concentration the mRNA level of *OsBZR1* and *DLT* was decreased [22, 23], suggesting *OsOFP8* behaves differently to *OsBZR1* and *DLT*.

OsGSK2 interacts with and phosphorylates the nuclear protein DLT [20], but we do not know whether the interaction with OsGSK2 causes subcellular distribution of DLT. OsGSK2 interacts with and phosphorylates OsOFP8, phosphorylation of OsOFP8 by OsGSK2 is required for its cytoplasmic localization. The phosphorylated OsOFP8 is exported from the nucleus and targeted for the proteasomal degradation in the cytoplasm. This phenomenon resembles the interaction between BIN2 and BZR1 [23, 30]. However, without BL treatment, BZR1 is located mainly in the cytoplasm [30], and BIN2 is distributed both in the nucleus and cytosol, as well as at the plasma membrane [31], but OsOFP8 is a nuclear protein. BR signaling converts phosphorylated BZR1 proteins to the dephosphorylated state [30], and BIN2 protein is rapidly depleted after 30 min treatment with 1 μM BL [32], whereas OsOFP8 protein level is increased under this treatment (Fig 2F). In rice, binding of 14-3-3 proteins to the phosthorylated OsBZR1 inhibits OsBZR1 function at least in part by reducing its nuclear localization [33]. Phosphorylated OsOFP8 may also adopt this mechanism to regulate its function. Indeed, there is a putative 14-3-3 motif in OsOFP8 protein (S3C Fig), providing a possibility that OsOFP8 may interact with 14-3-3 proteins to retain itself in the cytoplasm for degradation. Further studies are required to test the interaction between 14-3-3 and OsOFP8 and the possibility of cytoplasmic retention of phosphorylated OsOFP8 by 14-3-3 binding. Suppressing *OsBZR1* expression by RNAi leads to dwarf phenotype and reduced lamina joint bending [33], however, reducing expression level of *OsOFP8* by RNAi shows reduced lamina joint bending phenotype without dwarfism (Fig 1), suggesting *OsOFP8* may have other biological functions in addition to participating in BR signaling.

## Materials and Methods

### Plant material and growth conditions

The wild-type rice (*Oryza sativa* L.) plants Zhonghua 11 (*japonica* cv. ZH11) and OsOFP8 transgenic plants were grown in the experimental field at South China Botanical Garden in Guangzhou during the rice growing season. The angles between the leaf blades and the culms were measured with a protractor.

### TAIL-PCR

The *Osofp8* mutant was identified from T-DNA transformation. Genomic DNA of the *Osofp8* mutant was used as the template to amplify the flanking regions of the T-DNA insertion by high-efficiency thermal asymmetric interlaced PCR [24]. The primers are listed in S1 Table.

### Vector construction

For promoter analysis, a 1983 bp promoter sequence upstream of the translation start codon of *OsOFP8* was amplified by PCR. The PCR product was digested with *Eco*RI and *Nco*I, and inserted into the pCAMBIA1391z vector to generate the Promoter_*OsOFP8*_:*GUS* construct. Ten independent transgenic lines were obtained and showed β-glucuronidase (GUS) activity.

To overexpress *OsOFP8*, the full-length cDNA of OsOFP8 was PCR-amplified and inserted into the binary vector pCAMBIA1301-35S. The *OsOFP8* RNAi lines were generated by RNA interference, using a 280 bp fragment of the *OsOFP8* coding region. The fragment was inserted into an intermediate vector as positive and inverted directions, and then the whole cassette was cut out and inserted into the binary vector pCAMBIA1301-35S. The resulting constructs of overexpression and RNAi were introduced into *Agrobacterium tumefaciens* strain EHA105, respectively, and then transformed rice ZH11. The empty vectors were also transformed into ZH11 as controls.

Full-length OsOFP8 and OsGSK2 cDNAs were inserted into pBI221-YFP and pBI221-3HA vectors, respectively, to generate OsOFP8-YFP and 3HA-OsGSK2 constructs.

For BiFC assay, OsGSK2 and OsOFP8 coding sequence fragments were cloned into pSPYNE-35S and pSPYCE-35S vectors, respectively. The resulting constructs were co-transfected into protoplast cells, the transfected cells were incubated in dark for 12 h, and the fluorescence of YFP was observed.

### RNA extraction and quantitative RT-PCR

Total RNAs were isolated with Trizol reagent (Invitrogen) according to the manufacturer’s instructions. Total RNAs were pre-treated with DNase I, and first-strand cDNA was synthesized from 2 μg of total RNAs using oligo (dT)_18_ as primers (Promega, http://cn.promega.com/). The first-strand cDNA product was used as template in a 20 μL PCR reaction. For quantitative RT-PCR, SYBR Green I was added to the reaction system and run on a Roche real-time PCR detection system according to the manufacturer’s instructions. The melting curve was acquired at the end. The transcript data were calculated by Roche’s Software, and were normalized using *OsActin 1* as an internal control; the relative expression level was calculated by 2^-ΔΔCt^. Each experiment was performed with three replicates. The primers are listed in S1 Table.

### GUS staining

GUS staining was performed according to the method as described [34]. Different tissues of the Promoter_*OsOFP8*_:*GUS* transgenic plants were incubated in a solution containing 50 mM NaPO_4_ buffer pH7.0, 5 mM K_3_Fe(CN)_6_, 5 mM K_4_Fe(CN)_6_, 0.1% Triton X-100 and 1 mM X-Gluc at 37°C overnight. Images were taken under the stereomicroscope (Leica M165c).

### BR sensitivity assays

The lamina joint assay by the micro-drop method was performed as described previously [25]. A drop of ethanol (1 μL) containing 0, 10, 100 or 1000 ng of 24-epiBL, respectively, was spotted onto the top of lamina of the seedlings which were germinated for 2 days and grown for 3 days at 30°C. Images were taken after 3 days of incubation with 24-epiBL, and the angles of lamina joint bending were measured. The lamina joint assay using excised leaf segments was performed as described previously [35]. Synchronous seeds after 2 days germination were selected and grown in the dark for 8 days at 30°C. The entire segments comprising 1 cm of the second leaf blade, the lamina joint and 1 cm of the leaf sheath were floated on distilled water for 24 h and then incubated in 2.5 mM maleic acid potassium solution containing 1 μM 24-epiBL for 48 h in the dark. Lamina joint angles were measured, this experiment was repeated three times with similar results.

### Protoplast transient expression assay and fluorescence microscopy

For transient expression assays, typically, 4×10^4^ mesophyll protoplasts were isolated from 4-week-old *Arabidopsis* seedlings. Isolation of protoplasts and PEG-mediated transfection were as described [36]. For transient expression analysis of *OsOFP8-YFP*, 10 μg of the plasmid DNA were used to transfect the protoplast cells. The transfected cells were treated with 10 μM BRZ for 12 h, and then treated with 1 μM BL for 0, 0.5, 1 h after the removal of BRZ by washing. To test the OsGSK2-mediated cytosolic translocation of OsOFP8, plasmid DNAs containing *OsOFP8-YFP*, NLS-mCherry, and *3HA-GSK2* were co-transfected into protoplasts. After 8 h incubation, the protoplasts were incubated with or without 10 μM MG132 for 1h. All transient transfection experiments were repeated at least three times with similar results. YFP and RFP fluorescence was observed with a confocal laser scanning microscope (ZEISS-510 Meta). The signal intensities of YFP and RFP were quantitatively determined using LSM Image Browser Rel. 4.0 software.

### Protein-protein interaction and phosphorylation analysis

For yeast two-hybrid analysis, *OsOFP8*, *OsBZR1*, *OsGAK2* and *DLT* were cloned into either pGBKT7 vector or pGADT7 vector, their combinations were tested for interaction. The reported gene assay was performed following the manufacturer’s instructions (Clontech). In addition, the truncated fragments of *OsOFP8* were also ligated into pGBKT7 vector for the analysis of protein-protein interacting sites.

For Co-immunoprecipitation analysis, *OsOFP8-YFP*, *YFP*, and *3HA-OsGSK2* were co-transfected into protoplasts in different combinations as indicated. After 8 h of incubation, total cell lysates from protoplasts were prepared in IP buffer (10 mM Tris-HCl pH 7.4, 150 mM NaCl, 0.5 mM EDTA, 0.2% Nonidet P-40, 5% glycerol, 1 mM dithiobis and 1 x Complete Protease Inhibitor Cocktail) and were then incubated with GFP-Trap agarose beads (ChromoTek) for 4 h at 4°C in a top to end rotator. After incubation, the beads were washed four times with ice cold washing buffer (10 mM Tris-HCl, pH7.4, 150 mM NaCl, and 0.5 mM EDTA) and then eluted by boiling in reducing SDS sample buffer. Samples were separated by SDS-PAGE and analyzed by immunoblot using appropriate antibodies.

For phosphorylation analysis, Plasmids of *OsOFP8-YFP* and *3HA-OsGSK2* were co-transfected into protoplasts. After 8 h incubation, total cell lysates from protoplasts were prepared in IP buffer (10m MTris-HCl pH 7.4, 150 mM NaCl, 0.5 mM EDTA, 0.2% Nonidet P-40, 5% glycerol, 1 mM dithiobis, 1 × Phosphatase Inhibitor Cocktail, and 1 x Complete Protease Inhibitor Cocktail) and were then incubated with GFP-Trap agarose beads (ChromoTek) for 4 h at 4°C in a top to end rotator. After incubation, the beads were washed four times with ice cold washing buffer (10 mM Tris-HCl, pH 7.4, 150 mM NaCl, and 0.5 mM EDTA) and then eluted by boiling in reducing SDS sample buffer. Samples were separated by SDS-PAGE and followed by immunoblotting with biotin-pendant Zn^2+^-Phos-tag (BTL-111) according to the manufacturer’s instructions (Western Blot Analysis of Phosphorylated Proteins-Chemiluminescent Detection using BiotinylatedPhos-tag).

### Protein preparation and immunoblot analysis

Nuclear and cytoplasmic fractions in protoplasts were separated as described [37]. Protoplasts were lysed with a buffer (20 mM Tris-HCl, pH 7.0, 250 mM sucrose, 25% glycerol, 20 mM KCl, 2 mM EDTA, 2.5 mM MgCl_2_, 30 mM β-mercaptoethanol, 1 × protease inhibitor cocktail, and 0.7% Triton X-100) and fractionated by centrifugation at 3000 g for 15 min at 4°C. The supernatant was taken as the cytosolic fraction. The pellet was further washed with a resuspension buffer (20 mM Tris-HCl, pH 7.0, 25% glycerol, 2.5 mM MgCl_2_, and 30 mM β-mercaptoethanol) and reconstituted as the nuclear fraction. Each fraction was separated by SDS-PAGE and analyzed by Western blotting.

For total protein extraction from protoplasts, transformed protoplasts were harvested by centrifugation at 200 g for 3 min, followed by resuspension in lysis buffer containing 25 mM Tris-HCl, pH 7.5, 150 mM NaCl, 1 mM EDTA, and 1 × protease inhibitor cocktail. The protoplasts were further lysed by vortexing for 2 min. The total cell extracts were then centrifuged at 15,000 g for 30 min at 4°C; the supermatant were total protein and analyzed by immunoblotting with appropriate antibodies [38].

## Supporting Information

S1 Table Primers used in this study. (DOC)

S1 Fig Expression profile of *OsOFP8*. (A) qRT-PCR analysis shows that *OsOFP8* is expressed in various tissues examined. (B-J) GUS staining of different organs from the *PRO*_*OsOFP8*_:*GUS* transgenic lines. Native promoter of *OsOFP8* was fused to *GUS* gene to monitor the expression pattern of *OsOFP8*. GUS activity was detected in stem node (B), leaf sheath (C), stem (D), root (E), young leaf (F), and lamina joint (G). GUS activity was also detected in young spikelets (H), stamens and ovary (I), and in the embryo of seeds (J). Bars = 2 mm.
(TIF)

S2 Fig Characteristics of OsOFP8. (A) Schematic diagram of the N-terminal and C-terminal regions of OsOFP8. (B) The interaction between the N-terminal and C-terminal regions of OsOFP8, respectively, and the OsGSK2 were analyzed by yeast two-hybrid analysis. (C) Feature of OsOFP8 protein. Red letters indicate the predicted GSK3 phosphorylation sites. Green box indicates the putative 14-3-3 binding site. Underlines indicate the OVATE domain.
(TIF)

S3 Fig Subcellular localization of OsOFP8. YFP-OsOFP8 fusion (A) and OsOFP8-YFP fusion (B) were constructed to show the nuclear localization of OsOFP8. The nuclear marker NLS-mCherry was used as an indicator for the nucleus. Bars = 10 μm. (C) Western blotting to show the presence of co-transfected OsOFP8 and OsGSK2 proteins. N and C stand for the nuclear and cytoplasmic fractions, respectively. CBB represents Coomassie Brilliant Blue.
(TIF)

S4 Fig OsOFP8 transgenic lines and WT plants respond similarly to GA treatment. (A) WT (ZH11) and OsOFP8 overexpression and RNAi transgenic plants respond similarly to 10 μM GA treatment. (B) qRT-PCR analysis shows the expression levels of *GA20ox1* gene in WT, OsOFP8 overexpression (OE11) and OsOFP8 RNAi (RNAi 4) lines.
(TIF)
