# Correction: OVATE Family Protein 8 Positively Mediates Brassinosteroid Signaling through Interacting with the GSK3-like Kinase in Rice

**DOI:** 10.1371/journal.pgen.1006970

**Published:** 2017-08-22

**Authors:** Chao Yang, Wenjin Shen, Yong He, Zhihong Tian, Jianxiong Li

There is an error in panel A of Fig 3. Specifically, the yeast-two-hybrid (Y2H) results are duplicated for AD-DLT+BD-OsOFP8 and AD-OsBZR1+BD-OsOFP8 on the SD-Leu-Trp plate, and the positive control is missing from this panel. Please see the correct version of Fig 3 and its associated caption below.

**Fig 3:**
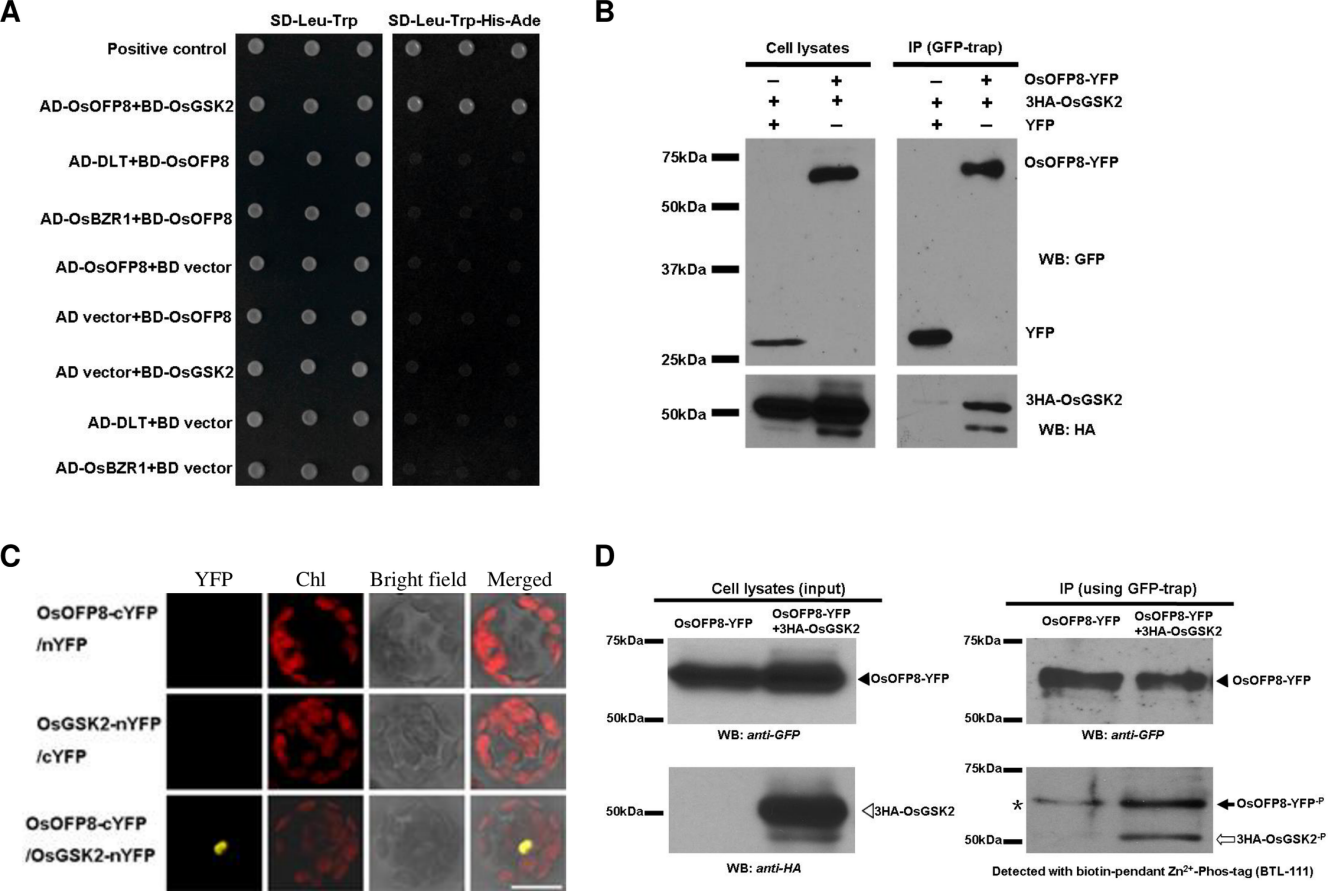
OsGSK2 interacts with and phosphorylates OsOFP8. (A) Yeast two-hybrid analysis for the interaction between OsOFP8 and OsGSK2,DLT, and OsBZR1. Co-transformed yeast clones were placed on SD dropout plates to detect the interactions. SD-Leu-Trp: synthetic complete medium lacking Leu and Trp for co-transformation detection. SD-Leu-Trp-His-Ade: synthetic complete medium lacking Trp, Leu, His, and Ade for interaction detection. Positive control, pGADT7-T (SV40 large T antigen) / pGBKT7-53 (murine p53). (B) Immunoprecipitation (IP) assay shows that OsOFP8 is associated with OsGSK2. Arabidopsis protoplasts expressing either YFP and 3HA-OsGSK2 or OsOFP8-YFP and 3HA-OsGSK2 were subjected to protein extraction. The Input (cell lysates) and IP were immunoblotted with indicated antibodies. WB:GFP indicates western blotting with GFP antibody. WB:HA indicates western blotting with HA antibody. (C) BiFC assay shows the interaction between OsOFP8 and OsGSK2. Chl means chlorophyll. (D) OsOFP8 phosphorylation analysis. Arabidopsis protoplast cells expressing OsOFP8-YFP only or OsOFP8-YFP with 3HA-OsGSK2 were subjected to protein extraction and then immunoprecipitated with either GFP antibody (WB: anti-GFP) or HA antibody (WB: anti-HA). Phosphorylation was detected with biotin-pendant Zn2+-Phos-tag (BTL-111). Black and white arrowheads indicate OsOFP8-YFP and 3HA-OsGSK2, respectively. Black and white arrows indicate phosphorylated OsOFP8-YFP^-P^ and 3HA-OsGSK2^-P^, respectively. * represents the internally phosphorylated OsOFP8-YFP.
