# Supplementary material for: OVATE Family Protein 8 Positively Mediates Brassinosteroid Signaling through Interacting with the GSK3-like Kinase in Rice
Source: PLoS Genet. 2016 Jun 22;12(6):e1006118. doi: 10.1371/journal.pgen.1006118 (PMC4917237; doi:10.1371/journal.pgen.1006118)
Supplement: S1 Table — (DOC) [file pgen.1006118.s001.doc]

**S1 Table.** Primers used in this study. Underlines indicate restriction enzyme sites.

| **Name** | **Forward (5’-3’)** | | **Reverse (5’-3’)** |
| --- | --- | --- | --- |
| **For TAIL-PCR** | | | |
| TAIL-1 | GGAACTGGCATGACGTGGGTTTCTG | |  |
| TAIL-2 | GTCCTGCCCGTCACCGAGATCTAACT | |  |
| TAIL-3 | TGAATGATCCGCTCCTGCATATGGG | |  |
| LAD1-2 | ACGATGGACTCCAGAGCGGCCGC(G/C/T)N(G/C/T)NNNGGTT | |  |
| **For plasmid construction** | | | |
| *OsOFP8ox* | CTTGGTACCCTCCAAGAACTCAACTCACT | | GAATCTAGACTAGAACTGGCAGGGGGACG |
| *OsOFP8*-*RNAi* | TAGGTACCTCTAGATCGCCCAACGCCTCCTCCAC | | GGGAAGCTTGGATCCGCTCCTGCAGCTGCTGCTGCT |
| *OsOFP8p*-*GUS* | GCGGAATTCGCTGGACATGAACACTCC | | GTCCATGGGGACAATGCCACTGGTGT |
| *YFP*-*OsOFP8* | TGCTCTAGAATGTCGGGCAGGTCGTCA | | AAGAGCTCCTAGAACTGGCAGGGGGACG |
| *Effector*-*OsOFP8* | TGCTCTAGAATGTCGGGCAGGTCGTCA | | GTTGGATCCCTAGAACTGGCAGGGG |
| *OsOFP8*-*AD/BD* | GTGGAATTCATGTCGGGCAGGTCGTCA | | GTTGGATCCCTAGAACTGGCAGGGGGA |
| *3HA*-*OsGSK2* | TAGACTAGTATGGACCAGCCGGCGCCGGC | | CCGCTCGAGTTAGCTCCCAGTATTGAAGA |
| *OsBZR1*-*AD* | CCGGAATTCATGACGTCCGGGGCGG | | CCCATCGATTCATTTCGCGCCGA |
| **For quantitative real-time RT-PCR** | | | |
| *OsOFP8* | CAGATGGTGGTGGAG | GTTGAGGGAGAGGAACTG | |
| *OsDWARF4* | ATGGTGTTGGTGGCGATTGGGGTGGTTG | ATGTTGTTCCGCCCCAGGATGTCCAGCA | |
| *OsD2* | AGCTGCCTGGCACTAGGCTCTACAGATCAC | ATGTTGTCGGAGATGAGCTCGTCGGTGAGC | |
| *OsGSK2* | GTCGCCTGCGACAAGAAGCA | AATTGTCCTCTTGGGCTCGCCG | |
| *DLT* | GTTAAGCGGGCGCATGACGA | TCGTCCCTGCAATGGATTGA | |
| *OsBZR1* | CCCAAGATCAGGAAGCCGGA | TCGCACTCCGGTATCGTGTC | |
| *OsActin* | CTCCCCCATGCTATCCTTCG | TGAATGAGTAACCACGCTCCG | |
