# Supplementary figures and images for: OVATE Family Protein 8 Positively Mediates Brassinosteroid Signaling through Interacting with the GSK3-like Kinase in Rice
Source: PLoS Genet. 2016 Jun 22;12(6):e1006118. doi: 10.1371/journal.pgen.1006118 (PMC4917237; doi:10.1371/journal.pgen.1006118)

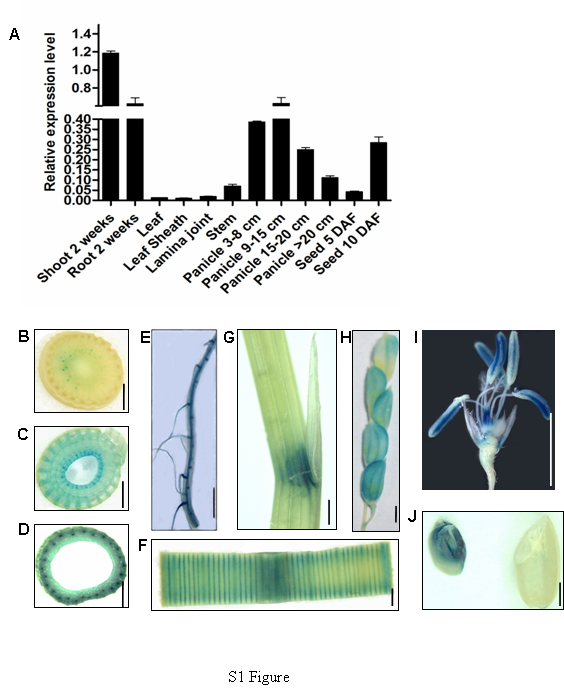

Supplement: S1 Fig — (A) qRT-PCR analysis shows that OsOFP8 is expressed in various tissues examined. (B-J) GUS staining of different organs from the PROOsOFP8:GUS transgenic lines. Native promoter of OsOFP8 was fused to GUS gene to monitor the expression pattern of OsOFP8. GUS activity was detected in stem node (B), leaf sheath (C), stem (D), root (E), young leaf (F), and lamina joint (G). GUS activity was also detected in young spikelets (H), stamens and ovary (I), and in the embryo of seeds (J). Bars = 2 mm. (TIF) [file pgen.1006118.s002.tif]

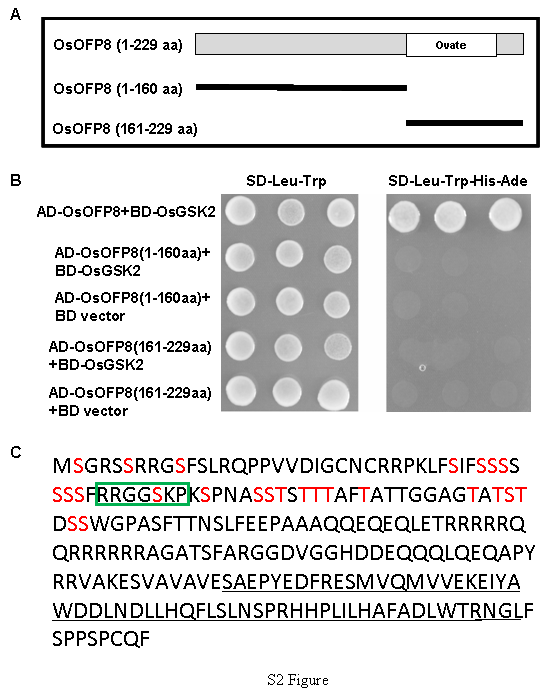

Supplement: S2 Fig — (A) Schematic diagram of the N-terminal and C-terminal regions of OsOFP8. (B) The interaction between the N-terminal and C-terminal regions of OsOFP8, respectively, and the OsGSK2 were analyzed by yeast two-hybrid analysis. (C) Feature of OsOFP8 protein. Red letters indicate the predicted GSK3 phosphorylation sites. Green box indicates the putative 14-3-3 binding site. Underlines indicate the OVATE domain. (TIF) [file pgen.1006118.s003.tif]

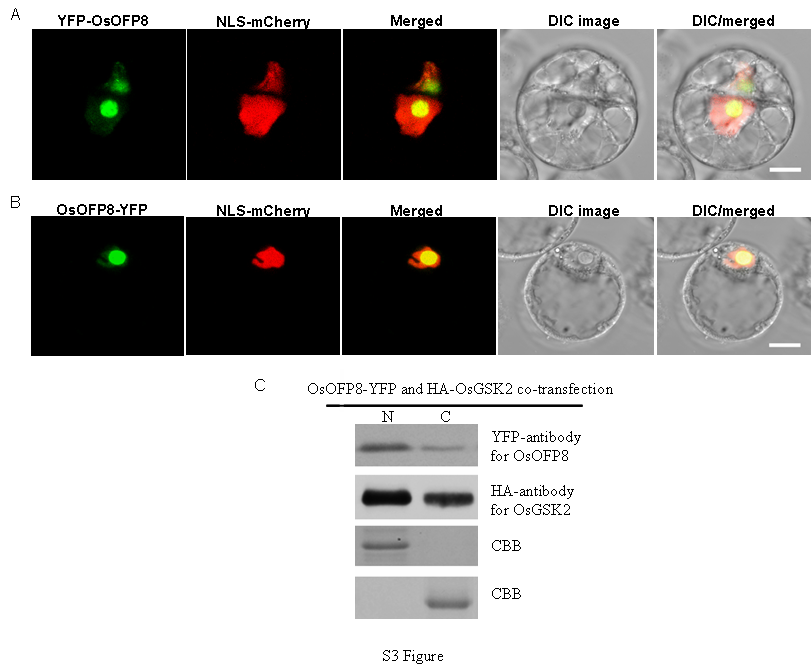

Supplement: S3 Fig — YFP-OsOFP8 fusion (A) and OsOFP8-YFP fusion (B) were constructed to show the nuclear localization of OsOFP8. The nuclear marker NLS-mCherry was used as an indicator for the nucleus. Bars = 10 μm. (C) Western blotting to show the presence of co-transfected OsOFP8 and OsGSK2 proteins. N and C stand for the nuclear and cytoplasmic fractions, respectively. CBB represents Coomassie Brilliant Blue. (TIF) [file pgen.1006118.s004.tif]

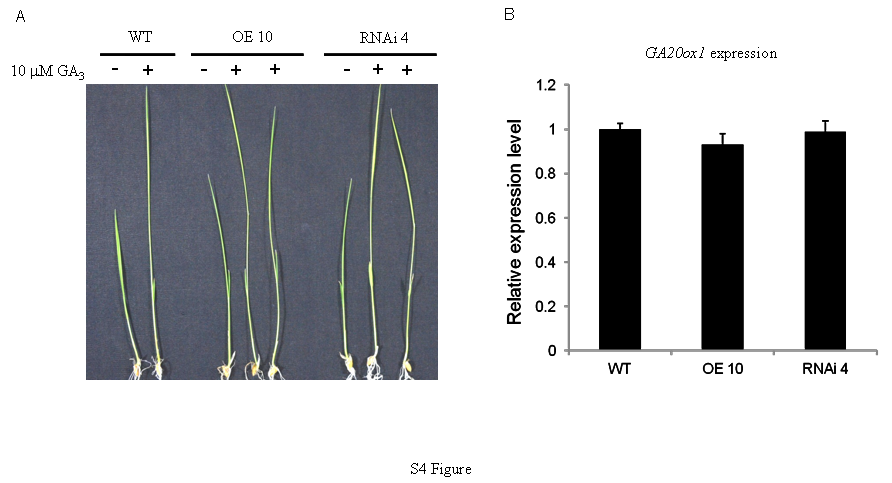

Supplement: S4 Fig — (A) WT (ZH11) and OsOFP8 overexpression and RNAi transgenic plants respond similarly to 10 μM GA treatment. (B) qRT-PCR analysis shows the expression levels of GA20ox1 gene in WT, OsOFP8 overexpression (OE11) and OsOFP8 RNAi (RNAi 4) lines. (TIF) [file pgen.1006118.s005.tif]
